# Graphene/Sulfur/Carbon Nanocomposite for High Performance Lithium-Sulfur Batteries

**DOI:** 10.3390/nano5031481

**Published:** 2015-09-01

**Authors:** Kangke Jin, Xufeng Zhou, Zhaoping Liu

**Affiliations:** Ningbo Institute of Materials Technology and Engineering, Chinese Academy of Sciences, No. 1219, Zhongguan West Road, Zhenhai District, Ningbo, Zhejiang 315201, China; E-Mail: jinkk@nimte.ac.cn

**Keywords:** Li-S battery, graphene, β-cyclodextrin, sulfur

## Abstract

Here, we report a two-step synthesis of graphene/sulfur/carbon ternary composite with a multilayer structure. In this composite, ultrathin S layers are uniformly deposited on graphene nanosheets and covered by a thin layer of amorphous carbon derived from β-cyclodextrin on the surface. Such a unique microstructure, not only improves the electrical conductivity of sulfur, but also effectively inhibits the dissolution of polysulfides during charging/discharging processes. As a result, this ternary nanocomposite exhibits excellent electrochemical performance. It can deliver a high initial discharge and charge capacity of 1410 mAh·g^−1^ and 1370 mAh·g^−1^, respectively, and a capacity retention of 63.8% can be achieved after 100 cycles at 0.1 C (1 C = 1675 mA·g^−1^). A relatively high specific capacity of 450 mAh·g^−1^ can still be retained after 200 cycles at a high rate of 2 C. The synthesis process introduced here is simple and broadly applicable to the modification of sulfur cathode for better electrochemical performance.

## 1. Introduction

Li-ion batteries are one of the most popular electrochemical energy storage systems at present, which have been widely used in areas from portable electronics to electric vehicles [1,2]. However, the gravimetric energy density of Li-ion batteries is known to be limited, which is far behind that of fossil fuels. The lithium sulfur (Li-S) battery, which possesses a much higher energy density than the Li-ion battery, has been increasingly attracting worldwide attention in the recent years [3,4].

Sulfur can yield a theoretical specific capacity of 1675 mAh·g^−1^ and a corresponding theoretical specific energy of 2600 Wh·kg^−1^ on the assumption of the complete reaction of Li with S to form Li_2_S [5]. Additionally, sulfur is abundant in various minerals, cheap, and environmental friendly. However, the development of Li-S batteries has met several challenges, such as the low electrical conductivity of sulfur, dissolution of polysulfides in electrolytes, and volume expansion of sulfur during discharge. These problems cause poor cycle life, low specific capacity, and low energy efficiency [6,7,8]. Thus, modification of sulfur cathode is necessary to overcome its intrinsic deficiencies. First of all, addition of electrical conducting agent is always required to improve the conductivity of sulfur [9]. Meanwhile, the size of sulfur should be minimized to shorten the diffusion length of both Li ions and electrons, as well as to ease the volume expansion of sulfur [10]. In addition, surface protection of sulfur is recommended to inhibit the dissolution of polysulfides [11].

Carbon materials, which have relatively high electrical conductivity and variable structures are extensively used in the modification of sulfur. Sulfur/carbon composites with diverse structures have been synthesized and exhibited remarkably improved electrochemical performance than pure sulfur [12,13,14,15]. Recently, graphene, as a new member of carbon materials, has started to play an important role in the application of energy storage due to its ultra-high electrical conductivity, large surface area, and flexible 2D nanostructure [16,17,18]. These features also endow graphene with great potential in the modification of sulfur cathode for high performance Li-S batteries, which has been broadly investigated in recent years.

One major function of graphene in the modification of sulfur is its high electrical conductivity, which can enormously improve electron transportation in sulfur cathode [19,20]. Meanwhile, the ultra-large specific surface area of graphene makes it a suitable substrate for the deposition of nanostructured sulfur to enhance kinetics of electrochemical reactions [21,22]. In addition, functionalized graphene (such as graphene oxide or graphene modified with organic groups) has strong interaction with sulfur, as proved in some reports, which is beneficial for immobilization of sulfur [23,24,25]. Recently, the potential of graphene or graphene oxide as a barrier to block the migration of polysulfides to the anode has been exploited and some exciting progress has been achieved [26,27]. Though numerous research experiments have demonstrated the remarkable advantages of multifunctional graphene in improving the electrochemical performance of Li-S batteries, sometimes, using graphene itself is not enough to fulfill the optimal modification of sulfur cathode. The combination of graphene with other functional components, such as carbon nanotubes [28,29], porous carbon [30], metal oxides [31], and polymers [32,33], have been extensively explored in recent years as an effective modification strategy to realize high specific capacity, good rate capability, and a long cycle life for Li-S batteries. It should be noted that the electrochemical performance of modified sulfur cathode is significantly dependent on its microstructure in addition to the materials used for modification. Elaborate structural design during the modification of sulfur has drawn a great deal of attention in recent years [29,34,35] and needs further exploitation for better electrochemical performance.

In this paper, a graphene/sulfur/carbon nanocomposite with a multilayer structure (named G/S/C hereafter), in which nanosized sulfur layers deposited on both sides of chemically reduced graphene sheets are then covered with amorphous carbon layers, is designed and successfully prepared. Such a unique structure realizes high conductivity, size miniaturization of sulfur, and surface protection of sulfur simultaneously, and, thus, gives rise to excellent charge/discharge performance. The G/S/C composite shows promising characteristics as a high performance cathode material for Li-S batteries.

## 2. Results and Discussion

**Figure 1:**
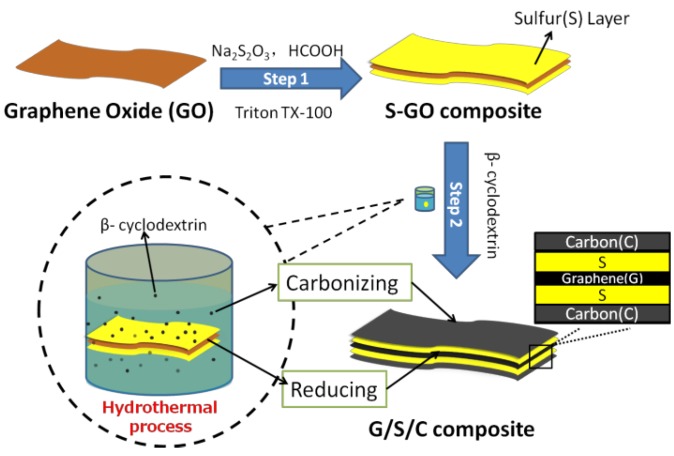
Schematic of synthesis steps for G/S/C composite and a proposed structure of the composite.

The experimental process to prepare G/S/C nanocomposite and its structure is illustrated in Figure 1. In the first step of the experiments, the solution reaction between Na_2_S_2_O_3_ and HCOOH in the presence of graphene oxide (GO) and nonionic surfactant of Triton TX-100 resulted in the formation of the sulfur-graphene oxide (S-GO) composite (Figure S1). The similar sheet-like morphology of the S-GO composite as that of the GO precursor and the strong signal of S in the energy-dispersive spectroscopic (EDS) spectrum of the samples indicates homogeneous deposition of thin S layers on the surface of GO, which can be ascribed to the affinity between S and GO aided by Triton TX-100. Then, the intermediate S-GO composite was mixed with β-cyclodextrin, and the mixture was hydrothermally treated at 180 °C for 12 h. The dehydration and carbonization of β-cyclodextrin under the hydrothermal condition generated thin carbon layers covering the surface of sulfur. In the meantime, graphene oxide was hydrothermally reduced. Finally, the G/S/C composite with a multilayer structure was produced. For comparison, graphene/sulfur nanocomposite (named G/S hereafter) was also prepared through the same reaction route without the addition of β-cyclodextrin.

**Figure 2:**
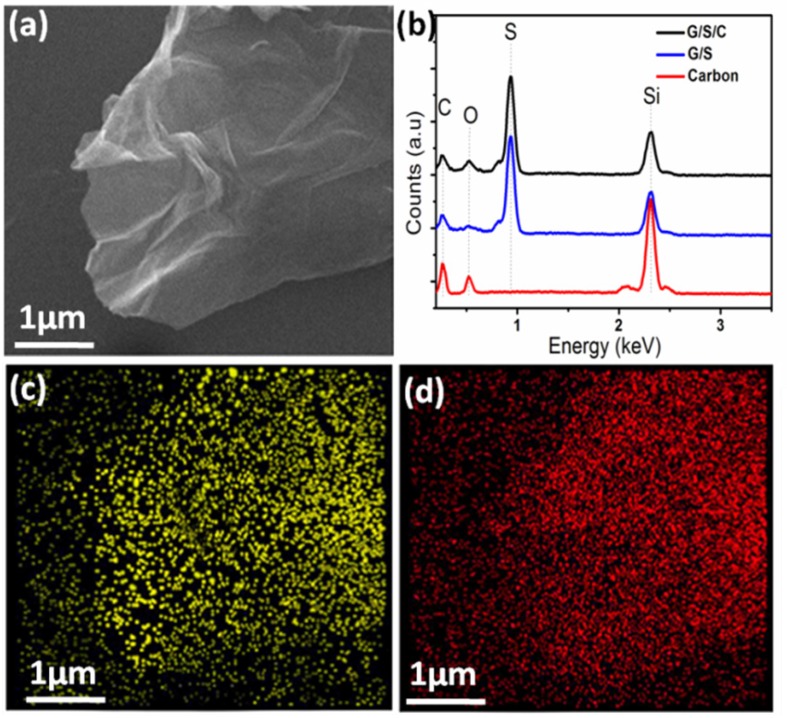
(**a**) SEM image of G/S/C composite; (**b**) EDS of G/S/C composite, G/S composite and carbon derived from β-cyclodextrin; (**c**) Elemental mapping of S in the region shown in (**a**); (**d**) Elemental mapping of C in the region shown in (**a**).

**Figure 3:**
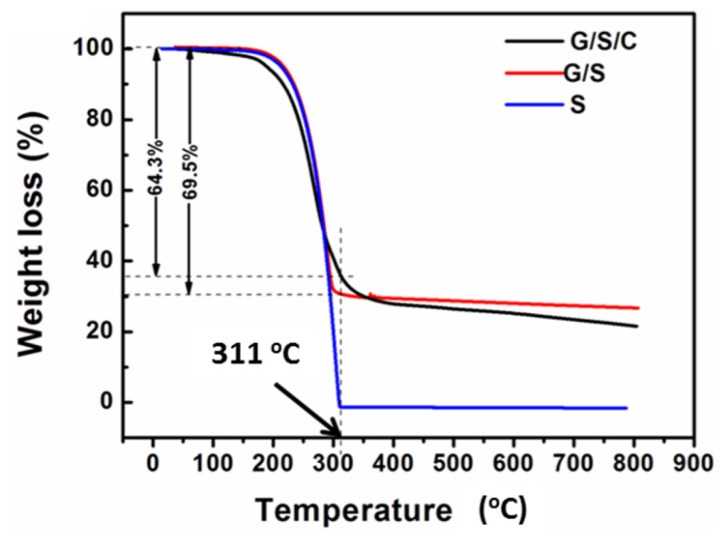
Thermogravimetric (TG) curves of G/S/C, G/S and pure S.

Figure 2a shows the scanning electron microscopy (SEM) image of the G/S/C nanocomposite. The sample has a typical sheet-like morphology, similar to that of graphene. The EDS of the sample (black line in Figure 2b) displays a strong signal of sulfur, suggesting a large content of S in the composite. The elemental mapping of the same composite sheet observed in Figure 2a reveals a homogeneous distribution of both sulfur and carbon over the entire sheet-like composite (Figure 2c,d). For comparison, the EDS microanalysis of G/S is also shown in Figure 2b (blue line). The spectra of G/S/C and G/S are almost identical except the signal intensity of oxygen. For G/S, the content ratio of O/C is much lower than that of G/S/C. The obviously higher content of O in G/S/C than in G/S is caused by the addition of β-cyclodextrin. The carbonaceous product obtained from hydrothermal treatment of pure β-cyclodextrin under the same experimental conditions as that to prepare G/S/C has a relatively high content of O, as revealed by EDS (red line in Figure 2b). Consequently, it can be deduced that carbon derived from β-cyclodextrin has been successfully incorporated into the final product. Thermogravimetric analysis (TGA) is used to determine the content of three components in the ternary composite of G/S/C (Figure 3). Pure S is completely evaporated at a temperature of 311 °C in N_2_. At the same temperature, G/S/C has a lower weight loss (64.3 wt.%) than that of G/S (69.5 wt.%), suggesting a lower content of S in G/S/C because of additional carbon layers from β-cyclodextrin. Taking into account that both pure graphene and pure carbon derived from β-cyclodextrin have the same weight loss of ~7 wt.% (Figure S2) at 311 °C, it then can be calculated that the weight content of sulfur, graphene and carbon in the G/S/C sample is approx. 62 wt.%, 30 wt.% and 8 wt.%, respectively.

**Figure 4:**
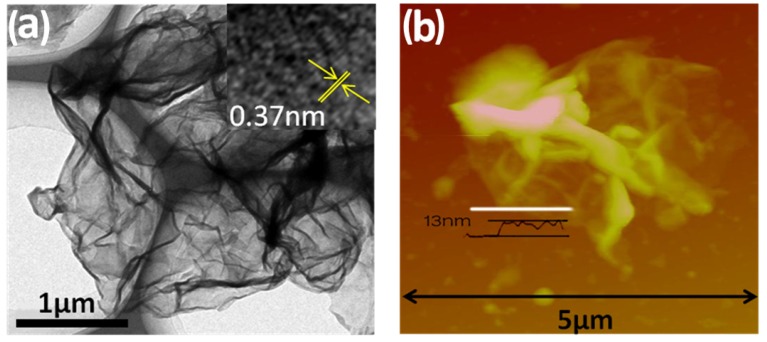
(**a**) TEM image; and (**b**) AFM image of G/S/C. Inset in (**a**) is the HRTEM image of G/S/C.

TEM image (Figure 4a) also reveals the sheet-like structure of the sample G/S/C. Though the contrast of the ternary composite is higher than that of pure graphene in TEM, sulfur and β-cyclodextrin derived carbon on graphene is still thin enough to allow penetration of an electron beam. In the high-resolution TEM image (inset of Figure 4a), the lattice fringes, with a distance of 0.37 nm, are clearly observed, which corresponds well with the crystal structure of sulfur. AFM image (Figure 4b) depicts the morphology and thickness of sample G/S/C. Wrinkles can be clearly seen for the sample deposited on Si substrate, as the sheet-like composite tends to crumple and fold easily. The measurement on the edge of one typical G/S/C flake indicates a mean thickness of ~13 nm. Taking the average thickness of single-layer reduced GO as ~1 nm, the thickness including sulfur and carbon layer on one side of graphene is estimated to be ~6 nm, suggesting ultrathin coating of sulfur and carbon. However, it should be mentioned that due to the non-thorough delamination of graphite oxide and/or inevitable restacking of graphene sheets during the synthesis of G/S/C nanocomposite, the graphene sheets in G/S/C may have two- or three-layers (2–3 nm in thickness), which implies that the thickness of sulfur layer should be even smaller. Combining all the data, G/S/C nanocomposite with a sheet-like morphology is believed to have a multilayer structure, *i.e.*, thin layers of sulfur deposited on both sides of reduced GO sheets are then coated by another layer of amorphous carbon derived from β-cyclodextrin on top.

**Figure 5:**
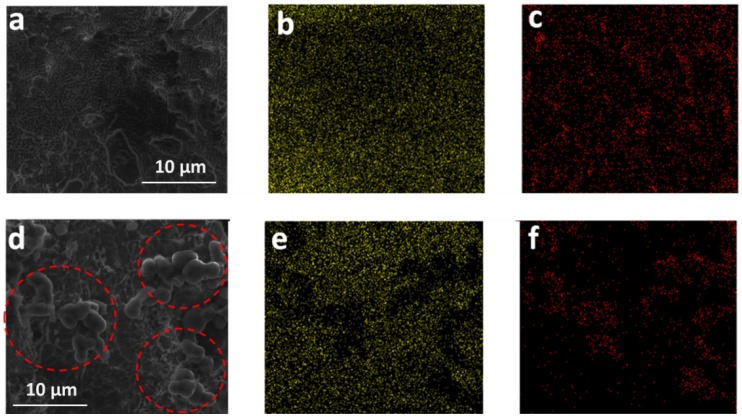
(**a**) SEM image of β-cyclodextrin derived carbon on the surface of sulfur; (**b**) Elemental mapping of S; and (**c**) elemental mapping of C in the region shown in (**a**); (**d**) SEM image of sucrose derived carbon on the surface of sulfur; (**e**) Elemental mapping of S; and (**f**) elemental mapping of C in the region shown in (d).

Graphene plays an important role in the formation of this multilayer nanocomposite. It acts as a sheet-like substrate for the uniform deposition of ultra-thin sulfur layers because of the strong interaction between oxygenous functional groups on GO and sulfur [23]. Only large and irregular sulfur particles are obtained in the absence of GO (Figure S3). The hydrothermal process is effective to reduce GO, which can be reflected by the comparison of the selected area electron diffraction patterns and FTIR spectra of GO before and after hydrothermal treatment as shown in Figure S4. The crystallinity can be enormously improved and the oxygen functional groups in GO are mostly removed after hydrothermal treatment. As a result, the electrical conductivity of reduced GO can reach ~10 S·cm^−1^, while GO itself is insulating. The choice of β-cyclodextrin as the carbon source is another key factor to obtain the multilayer structure. The β-cyclodextrin molecules contain both hydrophilic and hydrophobic groups, which can induce stronger interaction with sulfur comparing with hydrophilic biomass, such as sucrose [36]. To better demonstrate the advantage of β-cyclodextrin, hydrothermal treatment of the mixture of β-cyclodextrin and sulfur without graphene was carried out, and the same experiments using sucrose instead of β-cyclodextrin were also conducted for comparison. The weight ratio of the sulfur and carbon source is identical to that in G/S/C. SEM image and corresponding elemental mapping (Figure 5a–c) evidently display that a uniform distribution of element S and element C in the whole region, suggesting uniform coating of carbon layers on the surface of sulfur in the case of β-cyclodextrin. In contrast, when using sucrose as the carbon precursor, spherical particles with diameters of several microns (marked by dashed red circles) can be clearly observed on the surface of the sample (Figure 5d). Elemental mapping of S and C (Figure 5e,f) further confirms that such microspheres are carbon particles derived from sucrose through a hydrothermal process, which indicates that carbon from sucrose cannot form uniform coatings on the surface of sulfur. Even with the presence of graphene, a large number of carbon nanoparticles can still be observed in the ternary composite of graphene, sulfur, and sucrose derived carbon (Figure S5), rather than uniform carbon layers in the case of β-cyclodextrin. The above results prove the importance of β-cyclodextrin as the carbon source for the formation of multilayer structures, which can be mainly ascribed to its affinity to sulfur.

**Figure 6:**
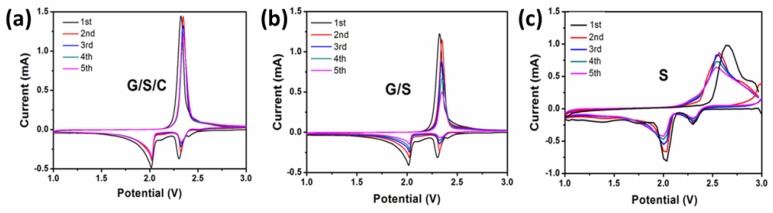
CV curves in the initial 5 cycles of Li-S cells using (**a**) G/S/C; (**b**) G/S; and (**c**) pure S as the cathode materials at 0.1 mV·s^−1^ in the potential window from 1 V to 3 V (*vs.* Li/Li^+^). 1 mol·L^−1^ lithium bis-trifluoromethanesulfonylimide + 0.1 mol·L^−1^ LiNO_3_ in 1,2-dimethoxyethane/1,3-dioxolane (1:1, by volume) solution was used as the electrolyte.

The electrochemical performance of the G/S/C nanocomposite as the cathode material was evaluated using coin cells with a lithium foil anode. For comparison, cells based on G/S without carbon coating and pure sulfur were also prepared using the same procedure. The cyclic voltammograms (CV) of cells based on three cathode materials are displayed in Figure 6. All three cells show typical redox peaks of sulfur. Comparing with pure sulfur, both G/S/C and G/S display much sharper redox peaks, indicating less polarization due to the presence of conductive carbon materials. The peak intensity of G/S/C reduces slightly within five cycles, whereas the intensity decreases dramatically for G/S, suggesting that the presence of carbon coatings on the surface is effective in improving the electrochemical stability of sulfur.

**Figure 7:**
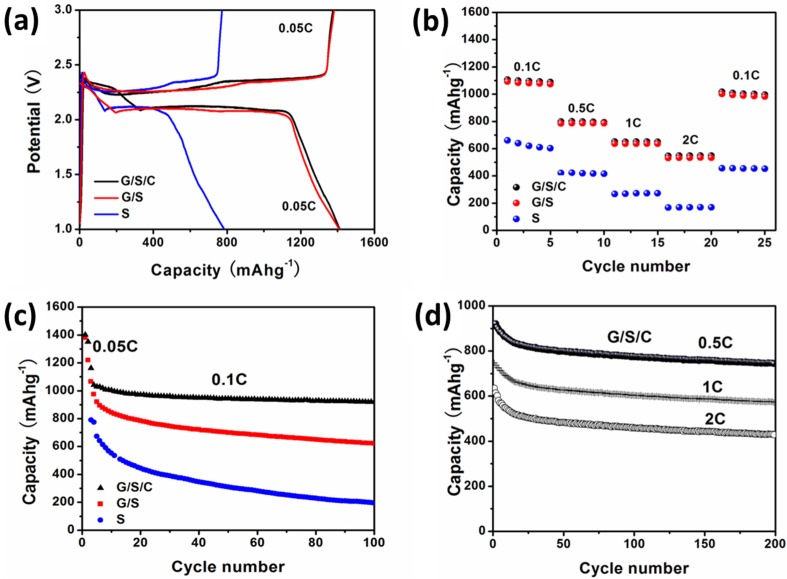
(**a**) Galvanostatic discharge/charge profiles at 0.1 C rate of cells using G/S/C, G/S and pure S as the cathode materials; (**b**) Reversible capacity *vs.* current density plots of cells using G/S/C, G/S and pure S as the cathode materials; (**c**) Cycling performance at a constant rate of 0.1 C after initial activation at 0.05 C for 2 cycles of cells using G/S/C, G/S and pure S as the cathode materials; (**d**) Cycling performance at rates of 0.5 C, 1 C and 2 C of cells using G/S/C as the cathode material. The specific capacity values are calculated according to the mass of S. 1 mol·L^−1^ lithium bis-trifluoromethanesulfonylimide + 0.1 mol·L^−1^ LiNO_3_ in 1,2-dimethoxyethane/1,3-dioxolane (1:1, by volume) solution was used as the electrolyte.

Figure 7a depicts the initial discharge and charge curves of three cells at the 0.05 C rate (1 C = 1675 mA·g^−1^) between 1.0 and 3.0 V. All the discharge curves show two plateaus in the voltage profile, which are consistent with the CV results. The charge/discharge curves for G/S/C and G/S are almost identical. Both samples can deliver a high initial discharge capacity of ~1410 mAh·g^−1^ and charge capacity of ~1370 mAh·g^−1^, corresponding to a high initial Coulombic efficiency of ~97%. As a comparison, pure sulfur only has initial discharge/charge capacities of less than 800 mAh·g^−1^, mainly due to its poor electrical conductivity. Another advantage of carbon modified sulfur over pure sulfur is the enhanced rate capability. As shown in Figure 7b, both G/S/C and G/S possess much higher reversible capacity than pure sulfur at elevated rates. A reversible capacity of ~550 mAh·g^−1^ can be reached for both G/S/C and G/S at a high rate of 2 C, while the capacity for sulfur is only 180 mAh·g^−1^ at the same rate. When the discharge rate was recovered to 0.1 C after cycling at high rates, a reversible capacity of ~1000 mAh·g^−1^ can still be retained. The above results evidently prove the important role of conductive carbon materials, especially graphene, with a high electrical conductivity in improving the electrochemical performance of sulfur.

Though G/S/C and G/S exhibit similar initial capacity and rate performance, their notable difference in cycling performance reflects the important role of the carbon layer on the surface of sulfur. As displayed in Figure 7c, all three cells were cycled at 0.1 C after an initial activation process at 0.05 C for two cycles. After 100 cycles, the reversible capacity of G/S/C remains at 900 mAh·g^−1^, which is 63.8% of the initial capacity. However, the reversible capacity of G/S fades to 567 mAh·g^−1^, only 44.4% of its initial capacity, much lower than that of G/S/C. Pure S exhibits the worst capacity retention of 24.7%. The apparently improved cyclic stability of G/S/C over G/S is undoubtedly ascribed to the carbon layer on sulfur, which is able to inhibit the dissolution of polysulfides and immobilize sulfur during charge/discharge cycling. The cyclic stability of G/S/C is further verified by cycling at higher rates. As shown in Figure 7d, it can maintain a reversible capacity of 800 mAh·g^−1^ after 200 cycles at 0.5 C, and the reversible capacity of 600 mAh·g^−1^ and 450 mAh·g^−1^ can still be retained after 200 cycles at 1 C and 2 C, respectively.

The electrochemical results shown above clearly indicates that the design of G/S/C nanocomposite with a multilayer structure, in which sulfur is embedded between graphene and thin carbon layers, is an effective approach to improve the electrochemical performance of sulfur. The graphene sheets not only raise the electrical conductivity of sulfur, but also facilitate the formation of ultrathin sulfur layers. The sulfur deposited on graphene has a thickness of less than 10 nm, which is beneficial for enhancing the electrochemical activity and utilization rate of sulfur, resulting in high specific capacity and good rate capability. The carbon coatings on the surface of sulfur, which are derived from β-cyclodextrin, mainly act as a protection layer to prevent the outflow of sulfur during long-time cycling, giving rise to excellent cyclic stability.

## 3. Experimental Section

### 3.1. Materials Synthesis

Graphite oxide was prepared using a modified Hummers’ method. The typical synthesis process is as follows: 6.0 g of KNO_3_ and 6.0 g of natural graphite (300 mesh) were added to 270 mL of concentrated H_2_SO_4_ (98 wt.%) at room temperature, and stirred for 10 minutes before slowly adding 36 g of KMnO_4_ into the mixture. The mixture was then heated to 40 °C and stirred for 6 h. Subsequently, under vigorous stirring, 480 mL of water was rapidly added and the slurry was continuously stirred at ~80 °C for another 30 min before 1200 mL of water and 36 mL of H_2_O_2_ solution (30 wt.%) were added sequentially to dissolve insoluble manganese species. The resulting graphite oxide suspension was washed repeatedly in water to be neutral. The graphite oxide became delaminated during washing, and graphene oxide powders were obtained by a freeze drying method.

One hundred and fifty milligrams of as-prepared graphite oxide were suspended in 60 mL of ultrapure water and then sonicated for 1 h to form a stable graphene oxide (GO) dispersion. Then, 3 g of Na_2_S_2_O_3_·5H_2_O was added to the GO dispersion in the presence of 0.5 mL of Triton TX-100. The mixture was sonicated for another 1 h and then directly titrated into 2 mol/L HCOOH solution at a rate of 30–40 drops/min until the pH reached 1.5 and stirred for another 0.5 h, and the intermediate nanocomposite of S-GO was obtained. Afterwards, 50 mg of β-cyclodextrin was added into the above suspension containing S-GO composites, and stirred for 1 h to dissolve β-cyclodextrin before the suspension was transferred into a Teflon-lined stainless steel autoclave with a volume of 50 mL. The autoclave was then placed in an oven and heated at 180 °C for 12 h. After cooling to room temperature, the black cake-like precipitate was isolated by filtrating and washing with deionized water and absolute ethanol several times. Finally, the as-prepared product was dried in vacuum at 60 °C for 10 h. Graphene/sulfur (G/S) nanocomposite was also prepared using the same experimental process except for the addition of β-cyclodextrin.

### 3.2. Material Characterizations

The morphology and structure of the materials were analyzed by a Hitachi S-4800 field emission scanning-electron microscope (SEM) and an FEI Tecnai G2 F20 transmission-electron microscopy (TEM) at an accelerating voltage of 200 kV. Atomic Force Microscopy (AFM) characterization was conducted by a Veeco Dimension 3100 V scanning probe microscope at ambient conditions using the tapping mode. Thermogravimetric analysis (TGA) was recorded in N_2_ atmosphere with a heating rate of 10 °C/min.

### 3.3. Electrochemical Measurements

CR2032-type coin cells were fabricated by sandwiching a porous polypropylene separator (Celgard 3501, Hoechst Celanese) between the cathode and a lithium metal foil (Cyprus Foote Mineral, 99.98%, USA) in an Ar-filled glove box. 1 mol·L^−1^ lithium bis-trifluoromethanesulfonylimide + 0.1 mol·L^−1^ LiNO_3_ in 1,2-dimethoxyethane/1,3-dioxolane (1:1, by volume) solution was used as the electrolyte. The cathode was prepared by mixing the active materials, carbon black, and polyvinylidene difluoride at a weight ratio of 80:10:10 in NMP solvent to form a slurry. The resultant slurry was uniformly spread via doctor blade on pure aluminum foil and dried at 60 °C for 48 h. The loading amount of active materials on aluminum foil is 2–3 mg·cm^−2^. Galvanostatic discharge and charge experiments of the coin cells were conducted using a cell testing system (LAND CT2001A, China) between cut-off potentials of 1.0 and 3.0 V. All of the electrochemical performance measurements were obtained at a constant temperature of 25 °C.

## 4. Conclusions

We synthesized a novel graphene/sulfur/carbon ternary nanocomposite with a multilayer structure as a cathode material for Li-S batteries. Ultrathin sulfur layers were embedded between graphene nanosheets and amorphous carbon layers obtained by hydrothermal treatment of β-cyclodextrin. Graphene nanosheets mainly enhance the electrical conductivity of sulfur, while amorphous carbon coatings on the surface play a critical role in suppressing the dissolution of polysulfides. The electrochemical properties of G/S/C nanocomposite were systematically investigated, and compared with those of G/S binary nanocomposite without carbon coating and also pure sulfur cathode. G/S/C exhibited the best electrochemical performance among three cathode materials, including high initial specific capacity, good rate capability, and cycling stability, which can be ascribed to its unique multilayer structure. This work offers a new strategy to the rational design and synthesis of sulfur cathode for high performance Li-S batteries.

## Supplementary Materials

Supplementary materials can be accessed at: http://www.mdpi.com/2079-4991/5/3/1481/s1.
