# Supplementary material for: Graphene/Sulfur/Carbon Nanocomposite for High Performance Lithium-Sulfur Batteries
Source: Nanomaterials (Basel). 2015 Sep 1;5(3):1481–92. doi: 10.3390/nano5031481 (PMC5304645; doi:10.3390/nano5031481)
Supplement: Supplementary file 1 [file nanomaterials-05-01481-s001.pdf]

Supplementary Information

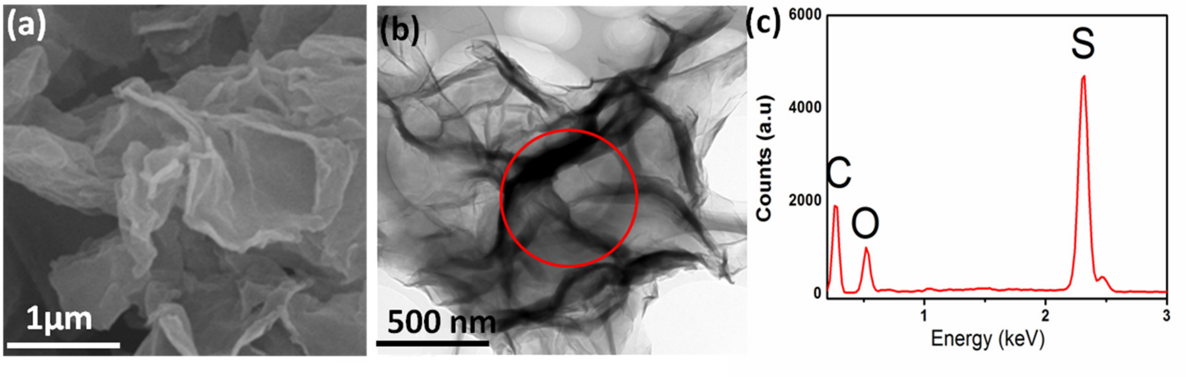

Figure S1. (**a**) SEM image; (**b**) TEM image and (**c**) corresponding EDS of the intermediate S-GO composite.

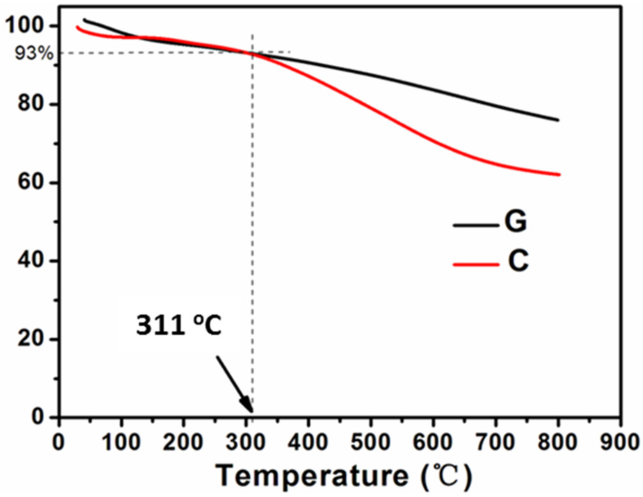

Figure S2. TGA curve of graphene (G) and carbon derived from β-cyclodextrin (C) recorded in N_2 with a heating rate of 10 °C/min.

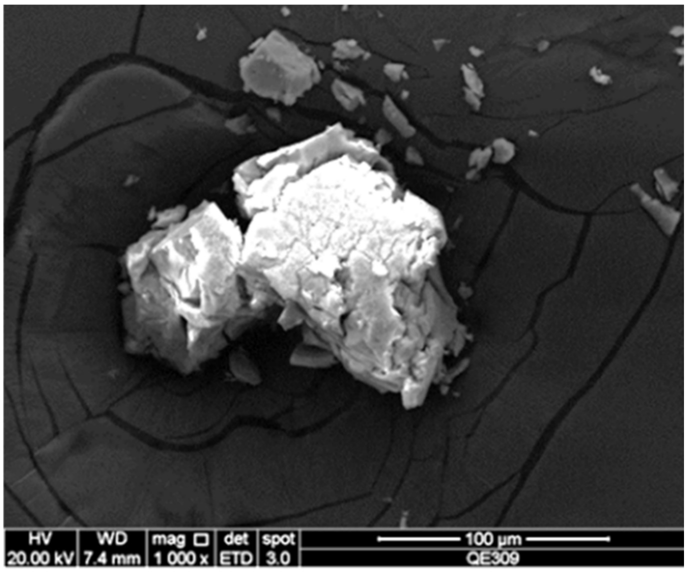

Figure S3. SEM image of sulfur particles prepared by the reaction between $Na_2S_2O_3$ and HCOOH without the addition of GO.

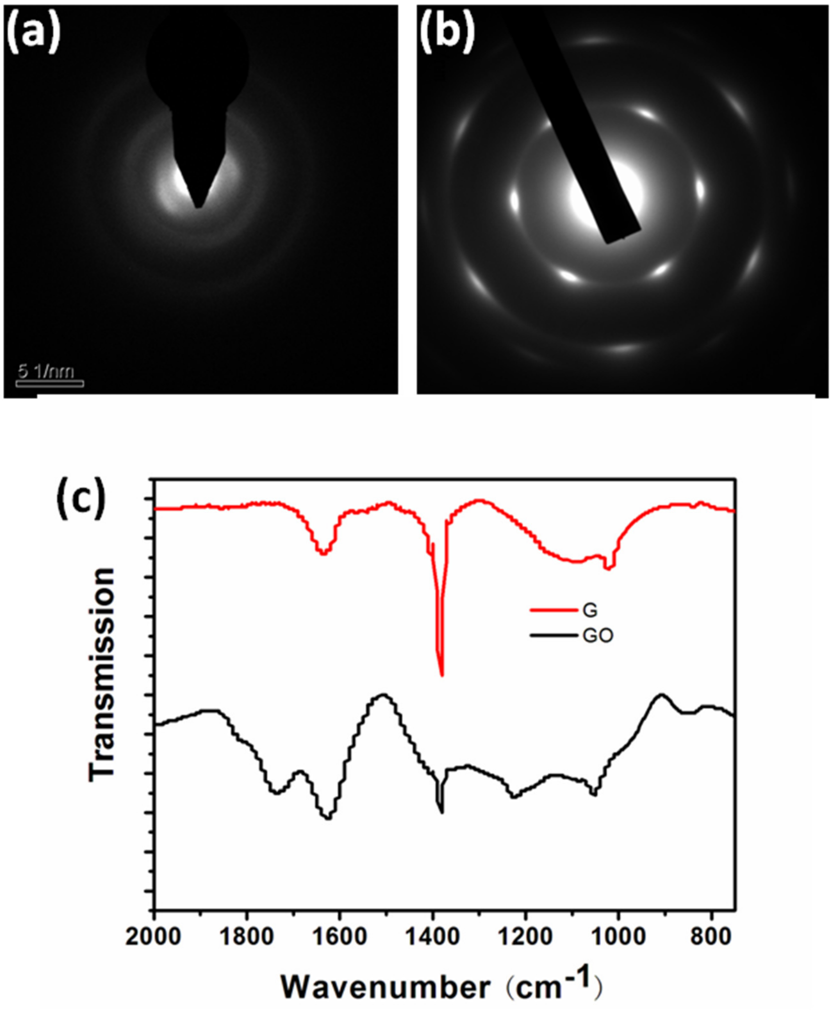

Figure S4. (**a**) SAED of GO; (**b**) SAED of reduced GO by hydrothermal treatment; (**c**) FTIR spectra of GO and reduced GO (G) by hydrothermal treatment. The bands at 1065 cm^{-1}, 1250 cm^{-1}, and 1751 cm^{-1} can be assigned to C–O stretching vibrations, C–OH stretching vibrations, and C=O stretching vibrations from carbonyl/carboxylic groups, respectively. Obviously, after hydrothermal treatment, these functional groups weaken significantly. Especially, the signals of C–OH and C=O almost disappear after hydrothermal process. The conductivity of graphene is proportional to the extent of reduction. Before reduction, conductivity of GO is only 0.001 S/cm, while reaches 10 S/cm afterwards.

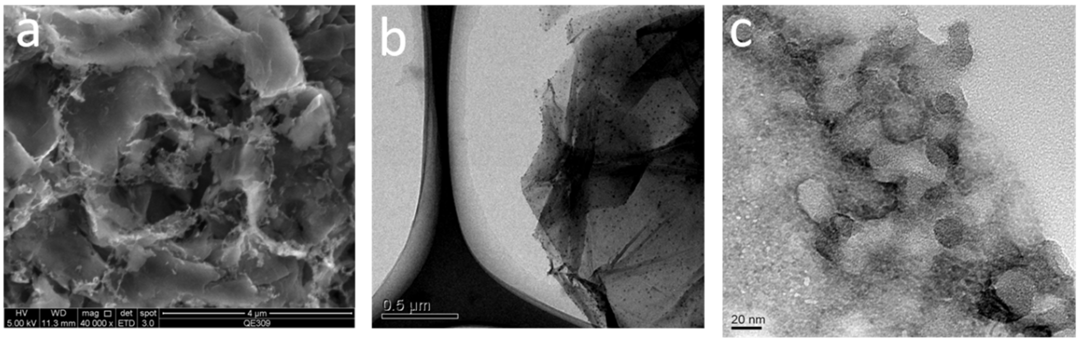

Figure S5. The SEM (**a**) and TEM (**b**,**c**) images of ternary composite of graphene, sulfur and sucrose derived carbon synthesized employing the same procedure as that of G/S/C except using sucrose instead of β-cyclodextrin. A large amount of amorphous carbon nanoparticles can be observed in this sample.

© 2015 by the authors; licensee MDPI, Basel, Switzerland. This article is an open access article distributed under the terms and conditions of the Creative Commons Attribution license (http://creativecommons.org/licenses/by/4.0/).
